# Can laboratory evaluation differentiate between coronavirus disease-2019, influenza, and respiratory syncytial virus infections? A retrospective cohort study

**DOI:** 10.3325/cmj.2021.62.623

**Published:** 2021-12

**Authors:** Ariel Ben Shimol, Shani Dahan, Nachshol Alon, Shelly Soffer, Keren Hod, Tal Brosh-Nissimov, Yehuda Shoenfeld, Amir Dagan

**Affiliations:** 1 Internal Medicine B, Assuta Medical Center, Ashdod, Israel; 2 Faculty of Health Sciences, Ben-Gurion University of the Negev, Be'er Sheva, Israel; 3 Department of Rheumatology, Wolfson Medical Center, Holon, Israel; 4 Sackler Faculty of Medicine, Tel Aviv University, Tel Aviv, Israel; 5 Department of Academy and Research, Assuta Medical Centers, Tel Aviv, Israel; 6 Infectious Diseases Unit, Assuta Ashdod Hospital, Ashdod, Israel; 7 Sheba Medical Center, The Zabludowicz Center for Autoimmune Diseases, Ramat Gan, Israel

## Abstract

**Aim:**

To identify clinical and laboratory parameters that can assist in the differential diagnosis of coronavirus disease 2019 (COVID-19), influenza, and respiratory syncytial virus (RSV) infections.

**Methods:**

In this retrospective cohort study, we obtained basic demographics and laboratory data from all 685 hospitalized patients confirmed with severe acute respiratory syndrome coronavirus 2 (SARS-CoV-2), influenza virus, or RSV from 2018 to 2020. A multiple logistic regression was employed to investigate the relationship between COVID-19 and laboratory parameters.

**Results:**

SARS-CoV-2 patients were significantly younger than RSV (*P* = 0.001) and influenza virus (*P* = 0.022) patients. SARS-CoV-2 patients also displayed a significant male predominance over influenza virus patients (*P* = 0.047). They also had significantly lower white blood cell count (median 6.3 × 10^6^ cells/μ) compared with influenza virus (*P* < 0.001) and RSV (*P* = 0.001) patients. Differences were also observed in other laboratory values but were insignificant in a multivariate analysis.

**Conclusions:**

Male sex, younger age, and low white blood cell count can assist in the diagnosis of COVID-19 over other viral infections. However, the differences between the groups were not substantial enough and would probably not suffice to distinguish between the viral illnesses in the emergency department.

Severe acute respiratory syndrome coronavirus 2 (SARS-CoV-2) is an RNA virus causing coronavirus disease 2019 (COVID-19). First identified in the Chinese province of Hubei in late 2019, COVID-19 was declared a global pandemic by the World Health Organization in March 2020 (1).

As of July 2021, there were more than 180 million confirmed COVID-19 cases and more than four million patients who died due to the disease complications (2). Moreover, the disease caused a substantial economic and social burden (3), and affected health care quality (4-7).

The diagnosis of COVID-19 is currently determined primarily by molecular methods and antigen tests (8,9). Radiographic diagnosis is possible as well (10,11). This practice often consumes valuable time and expensive equipment (12). There is a growing need to accelerate the diagnostic process by enabling point-of care diagnosis in various ambulatory settings, while keeping it accurate to ensure the necessary precautionary measures (13).

The clinical presentation of SARS-CoV-2 infection resembles that of other respiratory viruses, with predominant symptoms of fever, cough, fatigue, and dyspnea (14-17). Hematological abnormalities, including leukopenia, lymphopenia, and thrombocytopenia, are common among COVID-19 patients, as well as elevated levels of C-reactive protein (CRP), alanine aminotransferase (ALT), lactate dehydrogenase (LDH), and ferritin (14,15,18-21). Some of these inflammatory markers correlated with disease severity and mortality (22,23).

The influenza season of 2021 in the Northern hemisphere was relatively weak in contrast with predictions. Low to zero rates of influenza were detected in several countries. This was attributed to social distancing, masks wearing, and a reduced number of air travelers (24). Despite a growing number of vaccinated individuals (25), the emergence of new SARS-CoV-2 variants suggest that COVID-19 is here to stay. Seasonal viruses such as influenza virus and respiratory syncytial virus (RSV) could rebound in the following winter, with the loosening of restrictions.

Differentiating between COVID-19 and other respiratory viral illnesses on clinical grounds alone can be very challenging. These viral infections share similarities in the transmission route and symptoms (26-28). Several small studies attempted to delineate the differences in the clinical presentation of SARS-CoV-2 and influenza infections (29-31). In this study, we aimed to identify demographic and laboratory parameters that can assist in the early differentiation between SARS-CoV-2, influenza, and RSV infections in the emergency department.

## Methods

### Study design

This retrospective cohort study was conducted in Samson Assuta-Ashdod Hospital, Israel. Ethical approval was given by the institutional Ethics Committee, which waived the informed consent.

### Inclusion and exclusion criteria

The study enrolled all adult patients hospitalized in Samson Assuta-Ashdod hospital confirmed with a positive real-time polymerase chain reaction (RT-PCR) test to have SARS-CoV-2, influenza, or RSV during the period 2018-2020. Pediatric patients and two patients with dual-positive RT-PCR tests for RSV and influenza were excluded.

### Data collection

The following data were retrieved for each patient: age, sex, basic medical background, and laboratory analyses values documented at admission. Laboratory tests included white blood cells count (WBC), absolute lymphocyte count, platelet count, serum lactate dehydrogenase levels (DH), alanine aminotransferase (ALT), serum creatinine, C-reactive protein (CRP), albumin, and ferritin concentrations. Information regarding hospital length of stay was obtained.

### Statistical analysis

The normality of distribution was assessed with one-sample Kolmogorov-Smirnov test. Continuous data are presented as mean ± standard deviation (SD) or median (interquartile range), and categorical variables as the number of patients (percentage). For two-group comparisons, the Fisher exact test or chi square test were used for categorical variables, and the Mann-Whitney test or *t* test for continuous variables. For three-group comparisons the Fisher exact test or the chi square test were used for categorical variables, and the Kruskal-Wallis test for continuous variables.

Multiple logistic regressions were used to assess the relationship between SARS-CoV-2 and the laboratory parameters found in the univariate analyses to be significantly associated with SARS-CoV-2 infection (ie, WBC, ferritin, ALT), with age and sex adjustment. Odds ratios (OR) and their 95% confidence intervals (CI) were calculated. The significance level was set at *P* < 0.05 (two tailed). Statistical analyses were performed with SPSS, version 26 (IBM Corp., Armonk, NY, USA).

## Results

### Study population

The final analyses included 685 patients, classified into three groups: the RSV group with 79 patients; the influenza group with 393 patients; and the SARS-CoV-2 group with 213 patients (Table 1)

**Table 1:** Demographics and laboratory analyses in patients with severe acute respiratory syndrome coronavirus 2 (SARS-CoV-2), influenza virus, and respiratory syncytial virus (RSV)

	Patients with			P		
Characteristics	SARS-CoV-2 (n = 213)	RSV (n = 79)	Influenza (n = 393)	SARS-CoV-2 vs RSV	SARS-CoV-2 vs influenza	three-group comparisons
Age (y), median (interquartile range [IQR])	62.16 (44-77.5)	76.24 (70-86)	68.14 (57-83)	<0.001	<0.001	<0.001
Sex, n (%)				0.025	0.014	0.017
male	121 (56.8)	33 (41.8)	181 (46.1)			
female	92 (43.2)	46 (58.2)	212 (53.9)			
Comorbidities n (%)*	133 (62.4)	54 (68.4)	242 (61.6)	0.411	0.861	0.523
Laboratory analyses, median (IQR)						
white blood cells (10^6^/μL)	6.3 (4.8-8.6)	8.6 (6.9-11.2)	8 (5.7-11)	<0.001	<0.001	<0.001
absolute lymphocyte count (10^6^/μL)	1.1 (0.8-1.5)	1.0 (0.7-1.6)	0.9 (0.6-1.4)	0.371	<0.001	0.001
platelet count (10^3^/μL)	202 (163-247)	220 (181-287)	199 (158-254)	0.007	0.664	0.009
lactate dehydrogenase, (U/L)	438 (333-591)	402 (344-494)	409 (329-507)	0.199	0.035	0.098
creatinine (mg/dL)	0.9 (0.8-1.1)	1 (0.8-1.3)	0.9 (0.7-1.3)	0.163	0.224	0.328
alanine aminotransferase (U/L)	21 (14-35)	16.5 (12-23.25)	18 (13-28.75)	0.003	0.017	0.006
C-reactive protein (mg/L)	41 (10.9-100)	36 (17.8-86.6)	44.4 (19.7-85)	0.907	0.241	0.386
albumin (g/dL)	3.7 (3.4-4.1)	3.7 (3.5-4)	3.65 (3.3-4)	0.693	0.007	0.020
ferritin (μg/L)	329 (158-663)	210 (99-515)	245 (127-448)	0.045	0.017	0.024
Hospitalization length of stay (days) median (IQR)	4.9 (2.6-9.6)	4.2 (3.1-8.8)	3.5 (2.2-6.4)	0.752	0.001	<0.001

*Comorbidities include a prior diagnosis of at least one condition: diabetes mellitus, obesity, current malignancy, dementia, immunocompromised state (including organ transplant recipients, acquired immune deficiency syndrome, chronic use of corticosteroids of at least 20 mg prednisone per day or equivalent).

The study groups did not significantly differ in comorbidities (*P* = 0.523). Hospital length of stay was longer in the SARS-CoV-2 group (*P* < 0.001).

**Figure 1:**
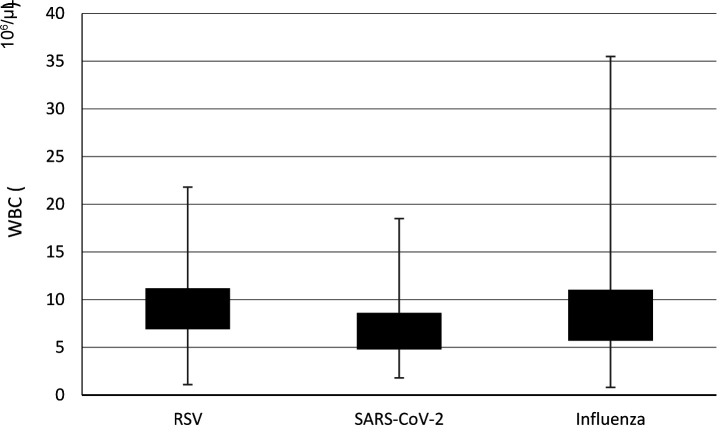
White blood cells (WBC, 10^6^ cells/μL) levels in the study groups. RSV – respiratory syncytial virus; SARS-CoV-2 – severe acute respiratory syndrome coronavirus 2.

In the univariate analyses, compared with the influenza and RSV group, SARS-CoV-2 patients had significantly lower WBC count (*P* < 0.001 and *P* < 0.001, respectively) (Figure 1) and significantly higher ALT (*P* = 0.017 and *P* = 0.003, respectively) and ferritin (*P* = 0.017 and *P* = 0.045, respectively).

The RSV group had higher platelet count (*P* = 0.007) than SARS-CoV-2 patients. SARS-CoV-2 patients had higher LDH (*P* = 0.035) and serum albumin levels (*P* = 0.007) than the influenza group.

Based on the univariate analysis, a logistic regression model was built (Table 2). The model included the variables significant in both comparisons in the univariate analysis: age, sex, WBC, ALT, and serum ferritin. SARS-CoV-2 patients were significantly more frequently male (*P* = 0.047), were younger (*P* = 0.022), and had lower WBC count (*P* < 0.001) than influenza patients. They also were significantly younger (*P* = 0.001) and had a lower WBC count than RSV patients (*P* = 0.001). SARS-CoV-2 were younger (*P* < 0.001) and had longer length of stay (*P* < 0.001) and lower WBC (<0.001) and platelet count (*P* = 0.002), and higher absolute lymphocyte count (*P* = 0.001), albumin (*P* = 0.020), ALT (*P* = 0.006), and ferritin (*P* = 0.024) than other two groups. Serum LDH and serum creatinine were not significantly different (*P* = 0.098 and *P* = 0.328, respectively).

**Table 2:** Logistic regression analysis comparing 1) severe acute respiratory syndrome coronavirus 2 (SARS-CoV-2) and influenza virus and 2) SARS-CoV-2 and respiratory syncytial virus (RSV)

	*P* value	Odds ratio	95% confidence interval
SARS-CoV-2 group (n = 213) vs influenza group (n = 393)			
age (y)	0.022	1.013	1.002-1.024
male sex	0.047	1.550	1.006-2.389
ferritin (μg/L)	0.557	1.000	1.000-1.000
alanine aminotransferase (U/L)	0.812	1.000	0.997-1.004
white blood cells (million/μL)	<0.001	1.152	1.085-1.224
SARS-CoV-2 group (n = 213) vs RSV group (n = 79)			
age (y)	0.001	0.963	0.942-0.985
male sex	0.133	0.567	0.270-1.189
ferritin (μg/L)	0.712	1.000	0.999-1.000
alanine aminotransferase (U/L)	0.891	1.001	0.987-1.015
white blood cells (million/μL)	<0.001	0.842	0.759-0.935

## Discussion

According to our study, in hospitalized patients with viral respiratory infections, SARS-CoV-2 infection was associated with male sex, younger age, lengthier hospitalization, and lower WBC counts in comparison with RSV and influenza infections.

Influenza virus and RSV are respiratory infections associated with significant morbidity and mortality during the winter season (32-34). The recent COVID-19 pandemic made the differentiation between respiratory viruses even harder. Moreover, different treatment approaches, the need for isolation precautions, and epidemiologic investigations require a rapid diagnosis of SARS-CoV-2 over other respiratory viruses.

In our study, the SARS-CoV-2 group was younger than the influenza and RSV group. This may be explained by two reasons. First, while all three diseases infect all age groups, a substantial share of young adults with COVID-19 present with severe symptoms (14,35). Second, lockdowns, collapsing health care services, disinformation, and higher anxiety and symptoms awareness probably encourage even people with mild symptoms to seek medical advice (36).

In addition, the SARS-CoV-2 group consisted mostly of men, while influenza and RSV groups exhibited female predominance. This supports the results of former studies, which reported a high male:female ratio among COVID-19 patients (14,37). Furthermore, male sex was a risk factor for severe disease and mortality in COVID-19 patients. Thus, one would expect a higher hospitalization rate for men (38-40). This might explain the higher proportion of male inpatients in our study.

In accordance with other studies, we found lower WBC count in SARS-CoV-2 patients compared with influenza and RSV (18). Other studies observed lymphocytopenia as a characteristic finding in COVID-19 patients (15,41). The younger age of our SARS-CoV-2 group even strengthens this claim, as WBC count tends to decline with age (42). Though absolute lymphocyte count was relatively low in our SARS-CoV-2 group, it was even lower in the RSV and influenza groups. Therefore, low lymphocyte count should not be considered specific in distinguishing COVID-19 from other viral infections. In this regard, the low WBC count in COVID-19 patients could not be explained solely by a low lymphocyte count. We assume that other components of WBC were also responsible for the total low count. Tanni et al (43) found significantly lower eosinophils levels in SARS-CoV-2 patients compared with influenza patients. Another study linked COVID-19 with lower monocyte counts (31). The immune dysregulation caused by COVID-19, as described by Ehrenfeld et al (44), could be another possible explanation for a lower WBC count in COVID-19.

Interestingly, although former studies found serum ferritin to be an important marker, as well as a prognostic factor in COVID-19, our study found no significant differences in ferritin levels or CRP, another inflammatory marker (22). This finding suggests ferritin to be a factor in other viral infections as well, as observed previously (45).

The hospital length of stay was longer for our SARS-CoV-2 patients compared with influenza and RSV patients. This finding, which indicates greater severity of COVID-19 compared with the other studied diseases, becomes even more noteworthy when one considers that SARS-CoV-2 patients were younger than patients hospitalized for the other viruses.

Several limitations could generate substantial bias in our data analysis. First, we collected data only from hospitalized patients; outpatients and home care patients were not included. This could affect the generalizability of the study, given that most COVID-19 patients have mild disease and many are asymptomatic (46,47). Second, the laboratory data were collected only at admission. These point values, however, could vary during the natural history of the disease. Third, though we included all inpatients with a diagnosis of either SARS-CoV-2, influenza, or RSV, the results of our study should be validated by a prospective study. Last, the demographics and clinical profile in this study do not reflect the general population. The participants were old and most of them had comorbidities. In addition, as mentioned in the first limitation, all of the participants suffered from moderate-severe disease. Whether our results can be extended to younger patients without comorbidities and with mild disease is currently unknown.

Although in our study SARS-CoV-2 infection was associated with male sex, younger age, lengthier hospitalization, and lower WBC counts in comparison with RSV and influenza, the differences between the groups were not substantial enough and would not suffice to distinguish between the illnesses in the emergency department. Molecular and other microbiologic diagnostic techniques, when available, still provide a more accurate and reliable diagnosis of COVID-19.
